# The Use of Machine Learning in the Diagnosis of Kidney Allograft Rejection: Current Knowledge and Applications

**DOI:** 10.3390/diagnostics14222482

**Published:** 2024-11-07

**Authors:** Tanja Belčič Mikič, Miha Arnol

**Affiliations:** 1 Department of Nephrology, University Medical Centre Ljubljana, Zaloška 7, 1000 Ljubljana, Slovenia; miha.arnol@kclj.si; 2 Faculty of Medicine, University of Ljubljana, Vrazov trg 2, 1000 Ljubljana, Slovenia

**Keywords:** kidney transplantation, rejection, diagnosis, machine learning, kidney biopsy

## Abstract

Kidney allograft rejection is one of the main limitations to long-term kidney transplant survival. The diagnostic gold standard for detecting rejection is a kidney biopsy, an invasive procedure that can often give imprecise results due to complex diagnostic criteria and high interobserver variability. In recent years, several additional diagnostic approaches to rejection have been investigated, some of them with the aid of machine learning (ML). In this review, we addressed studies that investigated the detection of kidney allograft rejection over the last decade using various ML algorithms. Various ML techniques were used in three main categories: (a) histopathologic assessment of kidney tissue with the aim to improve the diagnostic accuracy of a kidney biopsy, (b) assessment of gene expression in rejected kidney tissue or peripheral blood and the development of diagnostic classifiers based on these data, (c) radiologic assessment of kidney tissue using diffusion-weighted magnetic resonance imaging and the construction of a computer-aided diagnostic system. In histopathology, ML algorithms could serve as a support to the pathologist to avoid misclassifications and overcome interobserver variability. Diagnostic platforms based on biopsy-based transcripts serve as a supplement to a kidney biopsy, especially in cases where histopathologic diagnosis is inconclusive. ML models based on radiologic evaluation or gene signature in peripheral blood may be useful in cases where kidney biopsy is contraindicated in addition to other non-invasive biomarkers. The implementation of ML-based diagnostic methods is usually slow and undertaken with caution considering ethical and legal issues. In summary, the approach to the diagnosis of rejection should be individualized and based on all available diagnostic tools (including ML-based), leaving the responsibility for over- and under-treatment in the hands of the clinician.

## 1. Introduction

Kidney transplantation is considered the most effective treatment for patients with end-stage renal disease and is the most frequently performed solid organ transplantation [1]. Unfortunately, one of the most important limitations of kidney transplantation is kidney allograft rejection which limits long-term allograft survival [2]. To preserve the function of the kidney allograft, one of the most relevant factors is the timely diagnosis of rejection. The diagnostic gold standard for detecting rejection is a kidney biopsy. However, this is an invasive procedure that is time consuming and often stressful for the patient, with potentially serious complications requiring hospitalization, transfusions, or surgical/interventional procedures. In a large retrospective study of 2514 biopsies, the incidence of serious complications following a kidney transplant biopsy was 1.9% and it was significantly higher in patients undergoing an indication biopsy and it could occur up to 14 days after the procedure [3]. The need for a blood transfusion after the procedure was found in 4% of patients in another large nationwide French study [4]. Furthermore, despite the standardized approach in the form of the Banff classification, the assessment of allograft biopsies can often be difficult and time-consuming and is often inaccurate due to high interobserver disagreement [5]. In addition, the Banff classification is constantly evolving and becoming more complicated to interpret in everyday clinical practice [6], which can lead to misdiagnosis and potentially harmful consequences for the patient. Therefore, it is essential to develop novel diagnostic strategies to diagnose rejection and guide its prompt treatment.

Machine learning (ML) is a subtype of artificial intelligence (AI) that is becoming increasingly popular in many fields, including medicine. Compared to standard statistical methods, ML techniques can capture complex, non-linear relationships in data [7]. It is used in many areas of clinical medicine, especially in predicting cancer diagnoses, treatments, and outcomes [8]. ML can be (a) supervised, which means that prior knowledge of the output data is required, or (b) unsupervised, where the output data is unknown and the model discovers innate patterns within the data. Deep learning is described as a learning method in which the machine can automatically discover features needed for recognition/classification [9]. The basis of deep learning is a perceptron. If several perceptrons are connected to each other, this is referred to as an artificial neural network (ANN). A special form of an ANN that is used in image recognition and processing is called a convolutional neural network (CNN) [10]. In kidney transplantation, ML has been explored in several areas, namely in the prediction of early allograft function and survival, optimization of the dose of immunosuppression, estimation of post-transplant complications, forecasting live donor kidney function, and prediction of rejection including pathological and radiological evaluation of the allograft [11,12,13,14,15]. ML methods were also used to develop a non-invasive virtual biopsy system to predict day-zero biopsy findings based on 11 common donor characteristics. It is freely available as an online tool with the aim to assist clinicians in a timely and cost-effective manner [16].

The role of digital pathology in kidney transplantation using ML-based algorithms was recognized already in 2019 with the formation of the Banff Digital Pathology Working Group [17]. The group holds regular meetings with the aim of promoting the development, validation, and exchange of ML algorithms for image analysis in transplant pathology and improving the diagnostic accuracy and reproducibility of histopathological analysis [18]. In addition, on the occasion of the thirtieth anniversary of the International Banff Classification two years ago, AI- and ML-based algorithms were presented as key methods that could improve future Banff classifications and the need for further development and validation of ML methods in kidney transplant evaluation was emphasized [19].

Recently, it has been suggested that every nephrologist should be familiar with the basic concepts and potential use of ML in nephrology (and especially in transplantation), similar to statistical methods, including the basic understanding of the creation of predictive models [9]. Therefore, it is important to promote the development, use, and understanding of ML-based methods in kidney transplantation.

Our article reviews the current state of knowledge and advances in the diagnosis of kidney allograft rejection using various ML methods and suggests their use in daily clinical practice. Because the field of ML is growing so rapidly, it is difficult to keep up with all the relevant literature. During the preparation of our article, several new relevant studies on this topic were published, and we tried to include them in our review.

## 2. The Use of ML in the Diagnosis of Kidney Transplant Rejection

In recent years, several studies reported on the use of ML algorithms in the diagnosis of kidney transplant rejection and are presented in Table 1. ML algorithms were used for image analysis, predictive modelling, or both. The following text presents different diagnostic categories in which ML algorithms were used, namely histopathology, gene expression, radiologic evaluation, and standard-of-care parameters.

The diagnostic value of the studies presented was most commonly assessed using the performance metric area under the receiver operating characteristics curve (AUROC) or accuracy. These measures are influenced by the experimental design and study analysis and can sometimes be misleading depending on the test and validation datasets used in the study. Therefore, it is advisable to interpret performance statistics with caution. In our review, the results of performance statistics were briefly listed in Table 1 next to cited studies, but omitted from the main text to avoid possible misinterpretation.

### 2.1. Histopathology

CNN is an ML method frequently used in diagnostic medical imaging [47,48,49]. The first CNN-based study of transplant biopsies used in image analysis was performed in 2019 by Hermsen et al. and showed a significant correlation between visually scored elements of the Banff classification defined by a pathologist and network-based measurements. The study showed that there is considerable subjectivity in the assessment of transplant biopsies by pathologists (for example, counting of glomeruli) that could be eliminated by using a CNN-based model and it suggested that these algorithms should be further explored in the diagnosis of transplant biopsies [20]. Another study using a CNN-based model in image analysis was performed by Kim et al. [38]. The authors of the study detected C4d-positive or negative peritubular capillaries in giga-pixel immunostained slides of kidney biopsies. The proportion of C4d-positive peritubular capillaries in the immunostaining (C4d score) is one of the most important factors for the diagnosis of antibody-mediated rejection (ABMR). First, regions of interest (ROI) with sufficient tissue were defined in each slide and then categorized as feasible or non-feasible. C4d-positive peritubular capillaries were detected in feasible ROIs. Non-feasible ROIs were defined as artifacts or poorly stained areas that would limit interpretation, areas without peritubular capillaries, and scarred or infracted areas. The use of ML support improved the performance of the detection model compared to manual detection. Fifty and forty pixels of the magnified images showed the best performance in detecting C4d-positive and negative peritubular capillaries, respectively. The authors suggested that this system could evaluate the entire kidney biopsy sample and count all C4d-positive peritubular capillaries more accurately, which would help in the diagnosis of ABMR with lower interobserver variability. The same algorithm was further evaluated by Choi et al. who compared the pathologists’ diagnoses with the diagnostic performance of the algorithm and analyzed the associations of the algorithm with clinical data. The study was performed on 186 biopsies provided by two different transplant centers. Each slide was evaluated by three pathologists as well as the algorithm. The pathologists evaluated the slides independently and also jointly to obtain a consensus diagnosis. The algorithm showed similar diagnostic performance to the pathologists who reached a consensus diagnosis. The C4d scores assessed by the algorithm were significantly associated with notable microvascular inflammation, a higher detection rate of donor specific antibodies (DSA), and shorter allograft survival [41]. The main limitations of this study are the single-center nature of the training dataset that did not include protocol biopsies and the fact that some C4d-stained structures were very difficult to interpret so the consensus diagnosis was not completely reliable which might have influenced the performance of the algorithm.

Moreover, Kers et al. investigated the use of CNN in the detection of diseased kidney allograft biopsies. In this study, ML was used in image analysis and predictive modelling. This was a retrospective, multi-center, proof-of-concept study, using 5844 digital whole slide images of kidney allograft biopsies from 1948 patients. Single or serial CNNs were used to discriminate between normal or pathologic kidney biopsy images. The single CNN was trained to discriminate between three categories, namely normal kidney tissue, rejection, and other diseases. The two serial CNNs were trained in a two-step approach, first to differentiate between normal and pathological kidney tissue and then between rejection and other diseases. The study demonstrated the potential use of this automated method in the recognition of diseased biopsies by providing support to the pathologist, which could be particularly useful when a preliminary diagnosis is a priority [22]. The study was validated both internally and externally on an external real-world cohort of 101 patients. The main limitations of this study are that the data used for the training were from two Western European institutions and that some data such as initial nephropathy were missing.

Additionally, Becker et al. used a CNN-based model in image analysis and predictive modelling to differentiate between photographs of glomeruli from a biopsy with or without ABMR. The model showed great results in detecting diseased glomeruli. The model was trained using 279 images from glomerular transections on periodic acid–Schiff (PAS) stained slides from six biopsies with AMBR and six biopsies without AMBR. A heat map marked the areas of the biopsy image that were decisive for classification between ABMR and no ABMR, which could be useful to the pathologist in avoiding misclassification [21]. This was an example of a CNN model used for histologic classification. The number of samples used for training was small and there is no data on the validation process.

In a retrospective multi-center study by Labriffe et al., ML classifiers were constructed to recognize ABMR, T cell-mediated rejection (TCMR), and interstitial fibrosis-tubular atrophy (IFTA) from kidney biopsy samples [42]. ML was used in predictive modelling and used combined histologic (Banff 2013 criteria) and clinical data normally used to diagnose rejection. Clinical data included DSA, serum creatinine (SCr), and proteinuria at the time of the kidney biopsy. The training set consisted of two large European databases with 643 and 304 biopsies, respectively. The final diagnoses (ground truth) used in the training set were made by expert transplant physicians considering the patient’s medical history. Based on the variables used by the ML model, the study suggested that some parameters such as positive DSA and/or C4d positivity/negativity may be given less weight in the diagnosis of ABMR when specific histologic lesions are present, such as cg lesions. The algorithm was more sensitive in detecting ABMR than the Banff 2013 classification. Automatically assigned diagnoses can help pathologists standardize the interpretation of kidney transplant biopsies, which could be particularly useful in complex clinical situations. This was a large multi-center study with external validation on three datasets and similar performances of the ML model in each of them [42]. The ML model used in the study was extreme gradient boosting (XGB), a decision-based tree ensemble algorithm developed by Tianqi Chen. It is a supervised ML algorithm that can deal with missing values in the dataset and makes data preparation less time-consuming [50].

Another approach was shown in a study by Chauveau et al. [33]. The authors developed an ML-based algorithm that was used in image analysis and predictive modelling for the diagnosis of ABMR based on immunohistochemical analysis of three interferon-related proteins, tryptophanyl-transfer-ribonucleic acid (tRNA) synthetase 1 (WARS1), thymidine phosphorylase (TYMP), and guanylate-binding protein 1 (GBP1) in kidney allograft biopsies. The expression of these proteins reflects the endothelial damage present in ABMR. An ML model was created to diagnose ABMR for each of these three proteins in a two-step approach. First, each whole slide image was cropped into multiple square tiles and a CNN model was trained at the tile level for binary ABMR versus other disease classifications for each tile. Second, a random forest (RF) classifier was used to analyze the entire slide image based on the output of model 1 for all tiles. Internal validation was performed using a three-fold cross validation. Fifty-four kidney allograft biopsies were included in the study, seventeen biopsies with active ABMR and thirty-seven with differential diagnoses. All slides were interpreted by four nephropathologists (who did not know the diagnosis) based on the staining patterns in the microcirculation for each protein. Comparison of the ABMR diagnosis by nephropathologists or ML showed agreement for WARS1 and TYMP, but not for GBP1, where the ML strategy showed better sensitivity in detecting ABMR than nephropathologists (but less specificity). This study again showed that the use of ML models in different settings could help pathologists with the detection of ABMR. This could be particularly useful in C4d-negative cases without detectable DSA, where the diagnosis of ABMR is particularly difficult. Unfortunately, this was a small single-center study with only internal validation and variable performance, which is why the generalizability of the model is questionable. The RF that was used in the study was first introduced by Breiman et al. [51] and is considered one of the most accurate general-purpose learning techniques [52]. Random forests are a collection of classification and regression trees [53], which are simple models that use binary splits on predictor variables to determine outcome predictions. A major advantage of this method is its ability to process datasets with a large number of predictor variables [54].

### 2.2. Gene Expression

#### 2.2.1. Microarray-Based Molecular Diagnostic System

Changes in gene expression in kidney transplant rejection have been studied in detail as part of the Molecular Microscope Diagnostic System (MMDx) project using ML-based algorithms and aiming to define the molecular basis of rejection [55]. MMDx is currently available as a commercial test. Molecular testing is performed on an additional biopsy core that is stabilized in RNA*later* to prevent damage to the ribonucleic acid (RNA). In a first step, microarrays are used to evaluate the expression of messenger RNA (mRNA) in the biopsy. Currently, the expression of 19,462 unique genes is being measured [56]. Next, these measurements are interpreted using ML-derived algorithms. The biopsy sample is compared to a reference set of previously characterized biopsies. Rejection states and parenchymal injury as well as the probability of kidney transplant survival are then assessed. Since genes change their expression in a coordinated pattern, pathogenesis-based transcript sets associated with different types of rejection or injury have been created, based on the theory that one molecule plays multiple roles in various biological processes. The MMDx project, with its crude beginnings, has a history of nearly two decades and is constantly expanding its reference set and improving its ML-derived diagnostic algorithms [57].

TCMR and ABMR ML-based supervised classifiers, in particular linear discriminant analysis (LDA), were trained on the histologic phenotypes using microarray results from 403 kidney biopsies with the repeated ten-fold cross validation approach [58,59] and later validated prospectively in the INTERCOM study [60,61], in which TCMR and ABMR scores were prospectively assigned to 300 biopsies from six centers using the classifiers developed in the reference set and compared with the documented histology reports. The ABMR score correlated more strongly with allograft failure and early progression to failure than the conventional histology reports [61]. The TCMR score reclassified 26% of the biopsies. Discrepancies between the histology and TCMR score occurred mainly in cases with known limitations of the histologic assessment, such as biopsies with scarring and inflammation possibly due to other diseases [60]. Moreover, the prospective INTERCOMEX study from 10 different transplant centers confirmed the feasibility of the MMDx method in the real-time evaluation of kidney transplant biopsies and showed that MMDx was more often in agreement with the clinical judgment (87%) than the conventional histology (80%) (*p* = 0.0042). A classification tree that provided automated sign-outs predicted observer sign-outs very well [56].

In the following years, the method was further improved. An ensemble of ML classifiers was used to provide better estimates and more consistent results than a single classifier. This was done in a multi-centric study on 1208 indication biopsies [31]. Seven base classifiers were developed (TCMR, ABMR, i > 1, t > 1, g > 0, cg > 0, ptc > 0). For each of these classifiers, a ten-fold cross-validation was performed. In each fold, 12 different classifier algorithms were developed, that generated 12 test scores for each sample, with the final score based on the median of all 12 test scores. This was repeated for each of the seven base classifiers. The data was then used as input for the unsupervised archetypal analysis (AA). The final archetype model consisted of six archetypes (non-rejection (NR), TCMR, mixed rejection, and early-stage, fully developed, and late ABMR). All biopsy samples were assigned a score for each of the six archetypes. Assignment to clusters was based on the highest score within that biopsy. The results were visually presented in the principal component analysis (PCA).

A later study included 1679 kidney biopsies in the analysis by the same group [29]. It used 1208 biopsies from the previous study and an additional 471 biopsies (cohort 2) were added. The predictions for these 471 biopsies were generated using the algorithms from the previous study. Again, an ensemble of 12 different ML classifiers was used and an automated RF-based sign-out report was generated. The biopsies were divided in the two training sets and the test set from two cohorts (cohort one was used from the previous study). Predictions of ABMR and TCMR were made for each biopsy sample. An automated report was compared with an expert-formed diagnosis and histology interpreted locally according to the center’s standard of care and Banff guidelines. The study recognized that the ensembles of different ML classifiers provided more accurate diagnoses than individual ML classifiers. RF-based automated sign-outs showed a similar level of agreement with human experts (92% and 94% for predicting expert MMDx sign-outs for TCMR and ABMR, respectively). In contrast, there was significant disagreement with the histology. Disagreement with the histology was approximately 37%, with the potential to affect therapy present in approximately half of all cases, most commonly for TCMR assessment.

In other studies, the MMDx was used with a focus on the molecular phenotype of inflammation in areas of atrophy and fibrosis (i-IFTA) in kidney biopsies [37], the role of DSA in kidney biopsies with no evident rejection [34], and the correlation with donor-derived cell-free DNA (dd-cfDNA) in the Trifecta study [62].

Studies using MMDx are published on an ongoing basis covering different aspects of its use [63,64]. In a recent study, molecular classification algorithms were updated by training on genome-wide microarray measurements of 5086 biopsy samples from different studies. A new classification was developed with seven types of archetypes and the main objective to examine the subthreshold rejection activity [65].

The main limitations of the MMDx method are its centralization, the need for an additional biopsy core stabilized in RNA*later*, and the potential time delay between the time of biopsy and the MMDx results which are related to its centralization and may have clinical implications.

#### 2.2.2. Studies Based on Data from the Gene Expression Omnibus (GEO) Database

MMDx was the basis for the development of other molecular biopsy assessment platforms, for example the Banff-Human Organ Transplant (B-HOT) panel. This panel consists of 770 genes most relevant to the transplanted kidney and was developed by the Banff Molecular Diagnostics Working Group (MDWG) in association with the industry partner NanoString, Seattle, WA, USA [66]. In a study by van Baardwijk et al., a classifier was developed using the genes of the B-HOT panel to create a decentralized and multi-platform compatible molecular diagnostic tool (in contrast to MMDx) [30]. Three RF models were trained to predict rejection based on gene expression data from 1181 kidney transplant biopsies from one dataset from the GEO database. Nested cross-validation was used for each model. The three RF models used different sets of input features: the first model (B-HOT model) was trained on genes from the B-HOT panel, the second model was based on a sequential forward feature selection from all available genes in the selected database, and the third model (B-HOT+ model) was based on the combination of the first two models and included the six most predictive genes of the feature selection model (*CST7*, *KLRC4-KLRK1*, *TRBC1*, *TRBV6-5*, *TRBV19* and *ZFX*) that are not included in the B-HOT panel itself. All six most predictive genes play a role in the immune function. *CST7* is associated with inflammation and has been recognized as one of the signature genes of exTreg cells [67] that contribute to the creation of a pro-inflammatory environment [68]. The inclusion of six additional genes increased the performance of the model and thus the B-HOT+ model achieved the highest nested cross-validation performance and was the only model that was externally validated on a dataset from a different GEO database. External validation was performed for the NR and ABMR diagnosis but not for TCMR since the number of TCMR cases in the external validation dataset was low (*n* = 2). Further limitations of the study are its retrospective nature and the difference in the diagnosis of rejection between the training and validation groups (molecular and histological, respectively). The number of samples in the external validation set was overall low (*n* = 77).

Several other studies investigated the role of gene expression using the GEO database as the main source of genes. The GEO database is an international public repository that archives gene expression and epigenomics data generated using next-generation sequencing and microarray technologies [69] and has been known for 24 years [70]. It was established and is maintained by the National Center for Biotechnology Information (NCBI) and is publicly accessible at https://www.ncbi.nlm.nih.gov/geo/ (accessed on 30 March 2024). It continues to grow as gene expression studies in medicine become more common [71].

Dou et al. used gene expression profiles from kidney allografts as prognostic markers for allograft rejection based on data from eight datasets from the GEO database [23]. A combination of support vector machine (SVM) and recursive feature elimination (RFE) algorithms was used for gene selection. SVM is an important ML tool that operates as follows: a selected set of classified data is trained by the algorithm to obtain a group of classification models that can be used to predict the category of the new data. RFE is a feature selection model that can filter out relevant features and remove insignificant feature variables [72]. SVM-RFE has been commonly used for gene selection in several studies [73,74,75,76,77]. In the study by Dou et al., an immune-related prediction model RiskScore was created to predict allograft loss. Based on the SVM-RFE algorithm, five genes (*CXCL11*, *CCL4*, *CXCL10*, *IDO1* and *GBP2*) were selected for the prediction model. The expression of all five genes was upregulated in rejection tissues and was significantly higher than in NR tissues. Based on the median value of the RiskScore, the patients were divided into a high-risk and a low-risk group. All five genes were upregulated in the high-risk group. Kidney allografts in the high-risk group had significantly worse survival than those in the low-risk group. All five genes may play a role in rejection and are associated with innate immune cells [23]. The most studied is *CXCL10* that encodes C-X-C motif chemokine ligand 10. This is an interferon-gamma (IFN-γ)-induced chemokine that contributes to rejection by recruiting leukocytes. Its urinary levels are increased in cases of rejection, demonstrating its potential as a non-invasive biomarker for rejection. Unfortunately, as a general indicator of inflammation, it is not specific for rejection [78]. The study by Dou et al. was validated in GEO cohorts different from those used for training and in an independent single-cell dataset. The main limitations of the study are its retrospective nature and the fact that it was mainly based on information from the GEO databases.

Liu et al. performed RNA-sequencing (RNA-Seq) on biopsies from kidneys with stable function and biopsies with features of TCMR [43]. RNA was isolated from formalin-fixed, paraffin-embedded (FFPE) tissue. ML tools were used to develop prediction models to distinguish TCMR from stable kidney function using the top genes identified by differential expression analysis. The molecular signature discovery dataset consisted of five biopsies with TCMR and five biopsies with stable kidney function. The validation set consisted of 10 additional TCMR biopsy samples from the department’s archives and two GEO datasets containing a total of 703 biopsies (external validation sets). A total of 421 differentially expressed genes were identified, and a combination of the top 33 genes was selected to achieve the highest Youden index. The LDA and RF methods were superior to the SVM method. LDA is a method that can easily handle cases where the within-class frequencies are unequal [79]. The main limitation of the study is a small sample size in the training and internal validation datasets.

Potential non-invasive biomarkers for tolerance after kidney transplantation were investigated by Fu et al. [27] that identified a feature set of five relevant genes (*HLA-DOA*, *TCL1A*, *EBF1*, *CD79B* and *PNOC*). Three publicly available genome-wide expression datasets of peripheral blood lymphocytes from 63 tolerant patients were used and 14 different ML models were compared for their ability to predict spontaneous kidney allograft tolerance. The best subset selection (BSS) regression model was the best performing ML model for predicting kidney allograft tolerance based on differential gene expression. BSS is an ML model that aims to identify a useful subset of predictors to achieve the highest predictive accuracy and performs better in high signal-to-noise regimes [80]. Downregulation of *EBF1* was an independent factor predicting allograft rejection and failure. In addition, the expression of *HLA-DOA* was significantly higher in tolerant patients compared to patients with stable kidney function [27]. Early B cell factor one (EBF1) is a transcription factor that plays an essential role in B cell-specific gene expression and B cell differentiation [81]. In tolerant kidney transplant recipients, *EBF1* expression was upregulated, indicating its potential role in B cell-mediated tolerance and kidney allograft survival [27]. In contrast, the role of human leukocyte antigen DO alpha (HLA-DOA) in kidney allograft tolerance is still unclear. Studies show that HLA-DOA inhibits B cell presentation of antigens [82]. Enhanced presentation of donor antigens by circulating B cells, on the other hand, predicts rejection [83]. This study had one of the largest sample sizes of tolerant kidney transplant recipient patients and used different types of cross-validation for the validation process that showed stable performance results. The main limitation was the difference in the time off-immunosuppression between tolerant patients from the two GEO databases (7 vs. 156 months) which might have influenced the results of differentially expressed genes [27].

Gene expression profiles from peripheral blood as a non-invasive marker of rejection have been investigated in various studies. In a study by Lu et al., least absolute shrinkage and selection operator (LASSO) regression and SVM analysis were used [28]. LASSO is a regression analysis method that performs variable selection and regularization with the aim to improve the prediction accuracy and interpretability of the model [84]. One dataset from the GEO database was used as the main source of genes and was split in a 60–40 ratio into the training and internal validation dataset. External validation was performed on the data from a different GEO dataset. Five genes were recognized that correlated with the diagnosis of AR (*CPA6*, *EFNA1*, *HBM*, *THEM5* and *ZNF683*). All five genes were associated with the activity of immune cells. The most critical gene recognized was *ZNF683. ZNF683* was also the most important gene in predicting allograft survival, with its higher expression predicting longer allograft survival. Immune cells, particularly regulatory T cells, resting NK cells, and CD8+ T cells, correlated positively with *ZNF683* expression [28]. Studies have recognized the role of *ZNF683* in the regulation of NK cell development and potential negative control of IFN-γ production [85] as well as in the control of inflammation [86]. Its role may lie in the maintenance of T cell numbers and the promotion of memory cells resident in donor tissue, which may protect the kidney from rejection [28]. The sample size of this study was rather small with 35 cases of AR and 58 non-AR cases in the training cohort and 62 cases in the internal validation cohort. The role of *ZNF683* in the prediction of AR should be further prospectively validated.

Another similar study based on gene expression from peripheral blood developed a diagnostic model to predict AR based on three genes (*TSEN15* (tRNA splicing endonuclease subunit 15), *CAPRIN1* (cytoplasmic activation/proliferation-associated protein-1) and *PRR34-AS1* (PRR34 antisense RNA 1) using LASSO regression [32]. The test was very good at discriminating AR and also showed potential value in discriminating TCMR. *TSEN15* and *CAPRIN1* were found to be negatively correlated with innate immune cells and positively correlated with T cell subpopulations, with patients at risk of AR having lower expression levels of these two proteins. Overexpression of *PRR34-AS1* correlated with AR. The biologic mechanisms underlying these three genes are still unclear and yet to be determined [32]. The main limitation of the study is the small sample size (51 AR and 24 NR cases were used for training from one GEO dataset and 31 AR and 28 NR cases were used for validation from another GEO dataset).

Transcriptomic data from the prospective multi-center Genomics of Chronic Allograft Rejection (GoCAR) study deposited in the GEO database [87] were used to train a proprietary AI algorithm in the development of a commercial risk assessment tool that uses next-generation sequencing to assess a 17-gene mRNA signature in peripheral blood to predict AR (Tutivia™) [46]. It categorizes kidney transplant patients with low or high risk of early AR (six months post-transplant). The test was validated in 151 patients in a prospective multi-center observational study [46]. The negative predictive value of the test for the prediction of AR was 0.79 with a cut-off value of 50 as a result of the risk score and the positive predictive value of 0.60 with an odds ratio of 5.74. The performance of the test was correlated with the results of surveillance or a for-cause biopsy using the Banff 2019 criteria [46]. The sample size in the validation study was rather small, which may have influenced the performance of the test that warrants further validation.

Other studies of peripheral blood gene expression in non-invasive rejection detection have used ML methods to analyze data and create predictive models [88,89], although some of them with discouraging results. In a recent prospective multi-center study, EU-TRAIN, conducted in 412 patients, 19 blood mRNA biomarkers and 4 non-HLA antibodies showed no additional benefit in detecting rejection in the first year after transplantation beyond the standard-of-care parameters [89].

Studies using gene expression profiling in the diagnosis of kidney rejection differ in study design, data analysis, the platform used for genetic testing, and the ML methods used, some of which are proprietary. Additionally, and consequently, the genes presented as important for the prediction/diagnosis of rejection differ from study to study. Therefore, it is difficult to compare these studies face to face, although the performance of some studies overlap despite the differences in study design [90]. Currently, tests based on gene expression in peripheral blood are not recommended as a reliable diagnostic tool for detecting rejection and should be further investigated in prospective studies [91]. Price and accessibility are other potentially limiting factors for their worldwide use.

### 2.3. Standard-of-Care Parameters

With the aim to avoid invasive and costly procedures to diagnose rejection, Jo et al. used three different ML models (XGB, elastic net, and RF) and logistic regression to predict early subclinical rejection based on standard-of-care clinical, laboratory, and immunological findings [45]. Diagnosis of early subclinical rejection was based on two-week protocol biopsy findings (Banff 2007 classification) and data from 987 patients treated at a single center. Thirty-one of the most common characteristics in the peri-transplant phase were included as predictors in the analysis (SCr, blood group, HLA mismatch, donor type, delayed graft function, warm ischemia time, cold ischemia time, etc.). The performance of the model was assessed internally using hold-out validation. The main variables identified by all models as risk factors for early subclinical rejection were HLA-II mismatch and induction agent. Logistic regression and elastic net performed similarly well, and better than the other two ML models. The main limitations of this study are lack of external validation and the fact that the study was conducted at a single center. The selection of risk factors included in the analysis was done by the researchers and not by deep learning methods that could have captured other potential risk factors for early subclinical rejection.

### 2.4. Radiologic Evaluation

In a study by Shehata et al., a computer-aided diagnostic (CAD) system for early AR was evaluated using diffusion-weighted (DW) magnetic resonance imaging (MRI) data, more specifically the apparent diffusion coefficients (ADC) [35]. DW-MRI detects water molecules in the kidney; in combination with capillary perfusion and water diffusion, ADC can be measured. A total of 100 patients from a single center were included in the study, 30 patients without rejection and 70 patients with AR, with diagnosis based on renal histology. DW-MRI was usually performed two weeks after transplantation, in patients with rejection it was performed just before kidney biopsy. Deep learning and auto-encoders (AE)s were used for training and classification to discriminate between AR and NR in kidney transplants. The classifier was tested with different structures. The network structure with two hidden layers (s1 = 50 and s2 = 5) demonstrated the highest accuracy as well as sensitivity and specificity in detecting AR. The main advantage of this method is its non-invasiveness and rapid diagnostic results compared to histology. The main disadvantage of this study is that the study database was rather small and non-diverse (data from a single center). Validation was done by a four-fold and ten-fold cross validation; there was no external validation.

This method was later further improved with a combination of clinical and radiological parameters [39]. A total of 56 kidney transplant patients were included in the study, which combined DW-MRI image markers and clinical markers as input for the CNN classifier. ADCs were extracted from DW-MRI and fused with clinical biomarkers (SCr and creatinine clearance). Kidney transplant status was defined as AR or NR. In this study, ADC values largely overlapped between the two groups, so it was not possible to clearly distinguish between the two groups based on this information alone. The addition of clinical biomarkers to the ADC significantly improved the performance of the model for predicting AR. Additionally, the study compared different ML algorithms and recognized the CNN-based system as the most accurate in this setting compared to SVM and stacked AE. This was a multi-center study conducted in the United States and Egypt, with validation using different types of cross-validation.

In another study by the same group, a novel CAD system called RT-CAD was developed [26]. The diagnosis of rejection was again based on a combination of radiological and clinical parameters. Radiologic parameters included ADCs and the amount of deoxygenated hemoglobin extracted from DW-MRI scans and blood oxygen level-dependent MRI (BOLD-MRI) scans, respectively. DW-MRI and BOLD-MRI are both methods that provide information about the anatomy and function of the kidney, with the important advantage that they do not require the use of a contrast agent. Clinical information included SCr and creatinine clearance. These integrated biomarkers were used as feeding information for stacked AEs. The study enrolled 47 patients who underwent an MRI and renal biopsy. The diagnostic performance of the RT-CAD system showed promising results. The study was again validated internally and the sample size was small.

In another recent study by Zhi et al. [44], multiparametric MRI in combination with clinical biomarkers was used in a model called RtNet+ to determine AR. This was a single-center retrospective study based on data from 252 kidney transplant recipients. The CNN was designed to differentiate between AR, stable renal function, and chronic allograft nephropathy. An MRI was performed within one week prior to a kidney biopsy. The final diagnosis was based on biopsy findings and clinical data or clinical data only. Unfortunately, not all patients underwent a kidney biopsy, which might have affected the accuracy of the final diagnosis and thus the performance of the model.

None of the above-mentioned studies based on an MRI had external validation and the sample sizes were rather small which limits their reproducibility in other populations. Additionally, diagnostic methods based on an MRI are limited by contraindications to MRIs (for example metallic artificial material, claustrophobia), availability of the MRI machine, and technical experience of the radiologist in renal imaging.

## 3. The Application of ML-Based Algorithms in Clinical Practice

Over the last few years, several ML-based studies have been designed to support the diagnosis of kidney allograft rejection; some of them with promising results. Probably the most advanced and widely used ML-based diagnostic method is MMDx, with biopsy-based transcript diagnostics becoming an integral part of the Banff classification for ABMR [92]. In addition, various ML-based methods are likely to play an important role in future Banff classifications [19]. Diagnostic methods based on gene expression in peripheral blood using AI algorithms to predict rejection offer a non-invasive alternative, but the current sub-optimal performance together with high price of the tests limit the enthusiasm for their everyday use. Improvements in these methods are probably underway. It is clear that the implementation of ML-based diagnostic methods into routine clinical practice is usually done with great reluctance. Apart from ethical, legal, and regulatory issues, new methods are usually validated against the current gold standard (Banff) which can sometimes unintentionally show their underperformance. Large prospective clinical trials are needed to assess the clinical impact of new methods in terms of long-term kidney allograft outcomes. Moreover, ML can improve the prediction of an outcome but cannot form data or new hypotheses autonomously without any human input [93]. The quality of such algorithms usually depends on the quantity and quality of the data used for training [10]. Multi-center data with large databases can reduce the risk of overfitting and improve the generalizability of ML models [94]. However, the quantity of data needed is sometimes difficult to assess as it depends on the quality of the input data and the complexity of the task. Training on datasets with missing or imprecise data can lead to construction of models with poor performance. Additionally, the interpretability of the ML models is sometimes difficult to assess (the “black box” problem) [10]. ML approaches for processing small and imbalanced data are thoroughly investigated, especially in the field of kidney transplantation, and show high classification accuracy and generalization potential [95]. Such methods using limited and unbalanced data, which are common in medicine, should be further investigated.

**Figure 1:**
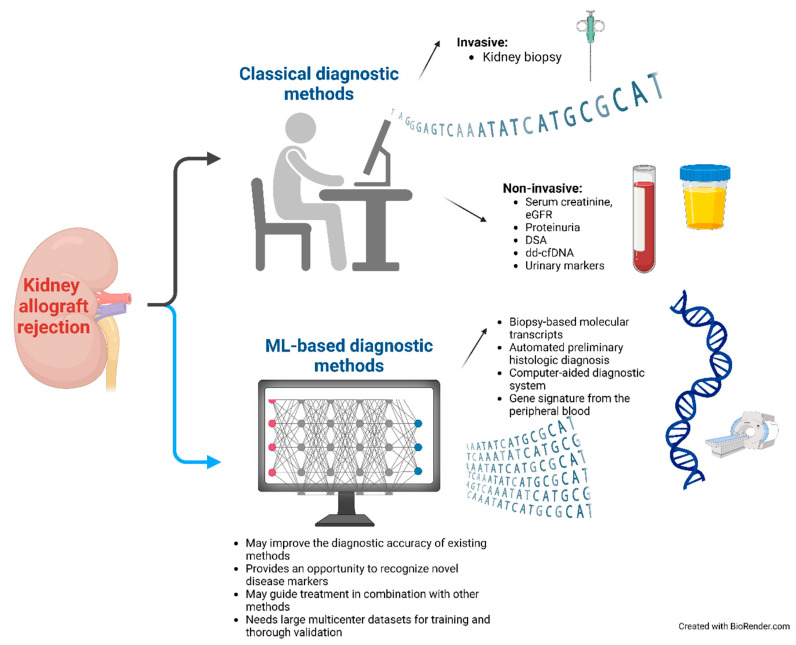
Diagnostic approach to kidney allograft rejection using classical and ML-based methods. eGFR, estimated glomerular filtration rate; DSA, donor-specific antibodies; dd-cfDNA, donor-derived cell-free DNA; ML, machine learning.

Currently, in kidney allograft rejection, it is probably best to use ML algorithms as a support for the clinician (as presented in Figure 1). For example, in histopathology, ML can perform specific tasks to assist the pathologist (e.g., counting C4d-positive peritubular capillaries) or provide a preliminary diagnosis based on histopathologic lesions present in the biopsy. Biopsy-based transcript diagnostics (MMDx or B-HOT panel) should be used in addition to histology, especially in the cases where the histologic diagnosis of a kidney biopsy is not entirely conclusive. Since inconclusive cases can sometimes be missed, the threshold for the biopsy-based transcript diagnostics should be low if it is logistically available and financially affordable. ML models based on radiologic and clinical data or gene signature in the peripheral blood may be useful in addition to other non-invasive biomarkers for rejection, especially in cases where invasive procedures such as kidney biopsy are contraindicated (as in patients receiving dual antiplatelet therapy). Most importantly, the approach to diagnosing rejection should be individualized for each patient. An integrative approach with all available diagnostic tools, including ML-based algorithms, should be used and all consequences of over- or under-treatment should be considered, which is always the responsibility of the clinician and cannot be delegated to an artificial structure.

## Figures and Tables

**Table 1:** Summary of studies that investigated ML in kidney transplant rejection over the last decade.

Reference	Focus Area	Type of Study	*n* and Type of Cases	Type of AI Algorithm	Main Results
Hermsen et al. [20]	Histopathology	The first study for multi-class segmentation of transplant biopsies and nephrectomy samples	40 transplant biopsies in the training dataset, 102 transplant biopsies from two centers and 15 nephrectomy samples in the test dataset	CNN	CNN-based classifications correlate with components of Banff
Becker et al. [21]	Histopathology	Retrospective study	279 images from 12 kidney transplant biopsies (6 biopsies with ABMR and 6 biopsies without ABMR)	CNN	Classification accuracy up to 91.3%
Kers et al. [22]	Histopathology	Retrospective, multi-center, proof-of-concept study	5844 digital whole slide images from 1948 patients	CNN	AUROC 0.87 for classifying kidney biopsy as normal vs. pathological, AUROC 0.75 for classifying pathological kidney biopsy as rejection or other diseases
Dou et al. [23]	Immune related genes	Retrospective study based on data from GEO database	8 datasets from the GEO database	SVM and RFE	Upregulation of 5 genes related to rejection and allograft loss, RiskScore predicted allograft loss (AUROC values of 1- and 3-year allograft survival 0.804 and 0.793, respectively)
Fang et al. [24]	Biopsy-based proteomic profiling	Proof-of-principle study	Biopsy samples from 15 patients	LDA, SVM, RF	329 proteins differentially expressed in TCMR, RF-based model predicted TCMR with 80% accuracy
Bae et al. [25]	Comparison of regression to ML models in predicting different transplant outcomes, including 1-year acute rejection	Retrospective study based on data from the Scientific Registry of Transplant Recipients	Registry data from 133,431 adult deceased-donor kidney transplant recipients	GB, RF	Regression outperformed ML in predicting rejection
Shehata et al. [26]	Diagnostic performance of RT-CAD based on DW-MRI, BOLD-MRI, SCr and CrCl in predicting rejection	Retrospective study	Clinical, histologic and imaging data from 47 patients	ANN	93.3% accuracy, 90% sensitivity, and 95% specificity of RT-CAD in distinguishing between AR and NR, AUROC was 0.92
Fu et al. [27]	Prediction of spontaneous kidney allograft tolerance	Retrospective study based on data from GEO database	Genomic microarray data from 63 tolerant patients from the GEO database	14 different ML models, BSS was the most powerful model	Sensitivity 91.7% and specificity 93.8% in the test group, *EBF1* and *HLA-DOA* most important genes in kidney allograft rejection
Lu et al. [28]	Prediction of acute rejection	Retrospective study based on data from GEO database	3 datasets from the GEO database	LASSO and SVM	5 genes associated with AR, *ZNF683* with the highest predictive performance of AR (AUROC 0.641~0.906)
Reeve et al. [29]	Assessing rejection in kidney transplant biopsies using the MMDx	Prospective proof-of-concept study	Microarray measurements of gene expression in 1679 biopsies	12 different ML classifiers, the median was used as an ensemble score	Disagreement between histologic diagnoses and MMDx: balanced accuracy 78% for ABMR and 73% for TCMR
van Baardwijk et al. [30]	Open access system to diagnose rejection based on gene expression (panel of 770 genes)	Retrospective study based on data from the GEO database	Gene expression data from 1181 kidney transplant biopsies, 3 different models	RF	B-HOT plus model was the most accurate with AUROC of 0.965 and 0.982 for NR and ABMR, respectively
Reeve et al. [31]	Diagnosing rejection based on molecular phenotypes	Prospective study from 13 centers	Microarray data from 1208 kidney transplant biopsies	AA	32% discrepancy rate with histology, AA predicted allograft failure better than histology
Wang et al. [32]	Diagnosing AR based on gene expression in peripheral blood	Retrospective study based on data from the GEO database	Gene expression profiles of 251 renal transplant patients with biopsy-proven diagnosis	RF, SVM-RFE, LASSO	Diagnostic model based on three genes (*TSEN15*, *CAPRIN1*, *PRR34-AS1*) showed high accuracy in predicting AR (AUROC 0.925 in the validation cohort)
Chauveau et al. [33]	Prediction of ABMR based on immunohistochemical analysis of 3 proteins, WARS1, TYMP and GBP1	Retrospective single-center study	Kidney biopsies from 54 patients	CNN, RF	AUROC 0.89 (±0.02) for WARS1, 0.80 (±0.04) for TYMP and 0.89 (±0.04) for GBP1 in diagnosing ABMR versus other diagnosis
Madill-Thomsen et al. [34]	The relevance of DSA positivity in biopsies classified as NR	Data from the INTERCOMEX study	Microarray results from 1679 biopsy samples	12 ML algorithms	DSA positivity in NR biopsies associated with mildly increased expression of ABMR-related transcripts and decreased allograft survival
Shehata et al. [35]	CAD system for early AR detection using DW-MRI data	Prospective single-center study	100 patients with NR and AR with imaging, laboratory and histologic data	AE	Diagnostic accuracy to distinguish AR and NR was 94% to 97%
Pineda et al. [36]	Prediction of rejection based on mismatched non-HLA genetic variants	Prospective single-center study	27 kidney transplant recipients and 28 kidney donors	RF	65 non-HLA variants predictive of ABMR, 25 variants predictive of TCMR
Halloran et al. [37]	Frequency of rejection in i-IFTA by using histology and MMDx	Data from the INTERCOMEX study	234 indication biopsies from 189 patients	MMDx classifier algorithms	i-IFTA biopsies occurred later, showed more scarring, and had more ABMR, TCMR was not common in i-IFTA
Kim et al. [38]	Histopathology	Retrospective single-center study	380 kidney biopsies	CNN	Deep-learning-assisted labeling increased the performance of the detection model to recognise C4d positive/negative PTCs
Abdeltawab et al. [39]	Non-invasive diagnosis of acute rejection based on DW-MRI and clinical biomarkers	Multi-center study	56 renal transplant recipients	CNN	92.9% accuracy based on imaging and clinical biomarkers in distinguishing NR from AR with 93.3% sensitivity and 92.3% specificity
Kang et al. [40]	Prediction of AR based on gene expression data	Retrospective study based on data from the GEO database	Two datasets from the GEO database	ANN	The PI3K/AKT/MTOR pathway related to AR
Choi et al. [41]	Histopathology	Bicentric retrospective study	186 slides of renal allografts	CNN	ML algorithm showed similar diagnostic performance to pathologists
Labriffe et al. [42]	Histopathology	Retrospective multi-center study	Data from several independent datasets	XGB	A mean AUROC 0.95–0.97 for ABMR diagnosis, 0.91–0.94 for TCMR, >0.96 for IFTA
Liu et al. [43]	RNA-Seq for the diagnosis of TCMR in FFPE tissue	Proof-of-concept study	Discovery data from 10 patients	LDA, RF, SVM	Sensitivity of RF to diagnose TCMR up to 88%, specificity 100%
Zhi et al. [44]	Diagnosing rejection using multiparametric MRI	Single-center retrospective study	Clinical and MRI data from 252 kidney graft recipients	CNN	AUROC up to 0.745 combining clinical and MRI data
Jo et al. [45]	Risk assessment model for early subclinical rejection	Single-center retrospective study	Data from 987 patients	RF, XGB, elastic net	HLA II mismatch and induction agent important predictors of early subclinical rejection, AUROC 0.712 for elastic net prediction model
Bestard et al. [46]	Prediction of early AR based on Tutivia™—a peripheral blood gene expression signature	Multi-center observational prospective study with the aim to validate Tutivia™	Data from 151 kidney transplant recipients	Different proprietary ML algorithms	Tutivia™ + creatinine greater AUROC than creatinine alone to predict early AR, AUROC up to 0.69

ABMR, antibody-mediated rejection; AE, auto-encoders; ANN, artificial neural network; AA, archetypal analysis; AR, acute rejection; AUROC, area under the receiver operating characteristics curve; (BOLD)-MRI, (blood oxygen level-dependent) magnetic resonance imaging; B-HOT, Banff-Human Organ Transplant; BSS, best subset selection; CAPRIN1, cytoplasmic activation/proliferation-associated protein 1; CNN, convolutional neural network; CrCl, creatinine clearance; DSA, donor-specific antibodies; EBF1, early B cell factor 1; GEO, Gene Expression Omnibus; GBP1, guanylate-binding protein 1; DW-MRI, diffusion-weighted MRI; FFPE, formalin-fixed, paraffin-embedded; HLA(-DOA), human leukocyte antigen (DO alpha); i-IFTA, areas of inflammation in atrophy-fibrosis; IFTA, interstitial fibrosis-tubular atrophy; LASSO, least absolute shrinkage and selection operator; LDA, linear discriminant analysis; ML, machine learning; MMDx, Molecular Microscope Diagnostic System; NR, non-rejection; PRR34-AS1, PRR34 antisense RNA 1; PTCs, peritubular capillaries; RF, random forest; RT-CAD, renal transplant computer aided diagnostic; (RNA)-Seq, (ribonucleic acid)-sequencing; SCr, serum creatinine; SVM-RFE, support vector machine-recursive feature elimination; TCMR, T cell-mediated rejection; tRNA, transferRNA; TSEN15; tRNA splicing endonuclease subunit 15; TYMP, thymidine phosphorylase; WARS1, tryptophanyl-tRNA synthetase 1; (X)GB, (extreme) gradient boosting.

## Data Availability

No new data were created or analyzed in this study. Data sharing is not applicable to this article.
